# Effect of Acacia Gum, NaCl, and Sucrose on Physical Properties of Lotus Stem Starch

**DOI:** 10.1155/2014/564564

**Published:** 2014-12-11

**Authors:** Ritika Puri, Balmeet Singh Gill, Yogesh Khetra

**Affiliations:** ^1^Food Science and Technology Department, Guru Nanak Dev University, Amritsar, Punjab 143001, India; ^2^Dairy Technology Division, National Dairy Research Institute, Karnal, Haryana 132001, India

## Abstract

Consumer preferences in east Asian part of the world pave the way for consumption of lotus stem starch (LSS) in preparations such as breakfast meals, fast foods, and traditional confectioneries. The present study envisaged the investigation and optimization of additives, that is, acacia gum, sodium chloride (NaCl), and sucrose, on water absorption (WA), water absorption index (WAI), and water solubility index (WSI) of LSS employing response surface methodology (RSM). Acacia gum resulted in increased water uptake and swelling of starch; however, NaCl reduced the swelling power of starch by making water unavailable to starch and also due to starch-ion electrostatic interaction. Sucrose restricted the water absorption by binding free water and decreased amylose leaching by building bridges with starch chains and thus forming rigid structure.

## 1. Introduction

Lotus (*Nelumbo nucifera*) belongs to the family of* Nelumbonaceae,* all parts of which are edible in various forms. It is widely cultivated in China, India, Japan, and Australia [1]. It is generally consumed as vegetable; chiefly the stem part is processed in different forms such as roasted, pickled, dried, and fried. The plant exhibits multiple nutritional and medicinal properties, hence considered as a popular health food [2]. The alkaloid (liensinine) extracted from the stem is effective in treating arrhythmia [3], sunstroke, fever, dysentery, diarrhea, dizziness, and stomach problems [4]. The stem extracts also possess antiobesity [2] and antidiabetic attributes [5]. Its seeds find applications in folk remedies as a diuretic, cooling agent, antiemetic, and an antidote in the treatment of tissue inflammation and cancer [6]. Biochemically, the rhizomes are composed of proteins, fats, carbohydrates, and minerals and are a good source of energy [7].

Starch is considered to contribute to the textural properties of various foods and has several industrial applications as a thickener, stabilizer, adhesive, gelling, and water retention agent [8]. It is the basic ingredient in various foods obtained from cereals and root crops. Lotus is loaded with starch [9], which is commercially available in China and Japan having numerous industrial applications as thickening agent in food products. Man et al. [10] compared the physicochemical properties of starches from seed and rhizome of lotus. Seed starches showed significantly lower swelling power than rhizome starches. Gani et al. [11] characterized lotus stem starches purified from three lakes of India. Scanning electron microscopy of lotus stem starches revealed small rounded and typical oval shaped granules with a smooth surface. Swelling, solubility, and water absorption were improved with increasing temperature from 50 to 90°C.

Consumer preferences lead to the consumption of LSS in preparations such as breakfast meals, fast foods, and traditional confectioneries and also the utilization of LSS as potential food additives. Processes such as extrusion, baking, and pressure-cooking which employ heat-moisture treatment with pressure and shear have foremost applications in production of snack foods, ready-to-eat (RTE) foods, breakfast cereals, and porridge. High proportion of starch is employed in formulations involving gums, salts, and sugars as potential ingredients to carry out reaction with starch in presence of water subsequently modifying its physicochemical properties. Hence, this interaction between additives and starch is of immense concern to food scientists.

Hydrocolloids (gums) are utilized in food industries since they improve stability, modify textural profile, and reduce the retrogradation rate of the starch [12]. Mandala and Bayas [13] investigated the effect of xanthan gum on swelling power, solubility index, and granules status of wheat starch dispersion (2% w/w). According to swelling power values and granules dimensions at 75°C, xanthan addition enhanced swelling. Salt and sugar are the two vital ingredients utilized in food industries. Foods containing starches hold usual nature of changing the physicochemical properties frequently by addition of salt and sugar during food processing and storage. Rice starch with high content of amylopectin exhibits better swelling properties, while salt addition reduces it considerably [14]. Sugar affects color, flavor, dimension, hardness, and surface finish of the food product. Sugar tends to disperse the protein and starch molecules making the product fragile, thereby preventing the formation of a continuous mass [15]. The influence of sugar on water uptake of starches has also been studied [16]. Jin et al. [17] reported decreased water solubility index of corn meal on sugar addition due to inhibited degradation of the starch molecules in presence of sucrose. So far, there is no information on the effect of such additives on the physical properties of lotus stem starch.

Hence, the aim of this study was to determine the effect of additives, that is, acacia gum, NaCl, and sucrose on the physical properties such as water absorption (WA), water absorption index (WAI), and water solubility index (WSI) of lotus stem starch (LSS) employing response surface methodology (RSM).

## 2. Material and Method

### 2.1. Raw Material

Fresh lotus stem was procured from local market of Amritsar, India. Acacia gum, NaCl, and sucrose were used as additives in this study. Sucrose was procured from Sisco Research Laboratories Private Limited, Mumbai, acacia gum was procured from Merck Specialties Private Limited, and NaCl was procured from Loba Chemie Private Limited, Mumbai. All the chemicals and reagents used for analysis were of AR grade.

### 2.2. Extraction of Starch

Starch granules in native form were extracted using the method of Wei et al. [18] with slight modification. Lotus stems were washed and then peeled to scrape off the outer skin and the peeled stems were then cut into small pieces. The small pieces were then homogenized with ice-cold water in a home blender followed by squeezing of the homogenate through four layers of cheese-cloth by hand. The fibrous residue was blended and squeezed twice with ice-cold water to facilitate the release of starch granules from the fibers. The combined extract was filtered with 100 mesh sieves. Starch suspension was left overnight. The starch was then extracted by washing four times with distilled water and centrifuged at 3200 rpm for 10 min. The yellow gel-like layer formed on top of the packed white starch granule was carefully scraped off and discarded. The process of centrifugation separation was repeated until no dirty material existed. The isolated starch was dried in an air oven at 40°C for 24 hours. The starch was estimated to have 98% of purity.

### 2.3. Physical Properties

#### 2.3.1. Water Absorption (WA)

For determination of WA, Beuchat [19] method was followed with slight modifications. 2.5 g of sample (*W*_*s*_) was taken in 25 mL of distilled water in a preweighed test tube. Contents were stirred using vortex shaker occasionally with a holding period of 30 minutes and finally centrifuged at 3000 rpm for 15 minutes. The water absorption (WA) was calculated by the ratio of hydrated starch (*W*_aw_) and weight of samples (*W*_*s*_):

(1) $$\begin{array}{c} \text{WA}=\frac{W_{\text{aw}}}{W_{s}}\times 100\;\left(\text{g}/\text{g}\right). \end{array}$$

For samples containing acacia gum, NaCl, and sucrose, LSS powder was mixed with these three additives and the procedure was continued as it was described for pure starch suspensions.

#### 2.3.2. Water Absorption Index (WAI) and Water Solubility Index (WSI)

WAI and WSI were determined using a modification of the method of Leach et al. [20]. Starch dispersion of 2.5% was put in centrifugal tubes and heated in a water bath at temperature of 90°C for 15 minutes. In order to prevent granules' sedimentation during heating, stirring was applied periodically using glass stirrers. All tubes were covered with plastic covers in order to prevent water loss. After heating, samples were centrifuged (3000 rpm, 10 min). Precipitated paste was separated from supernatant and weighed (*W*_*p*_). Both phases were dried at 105°C for 24 h and the dry solids in precipitated paste (*W*_dp_) and supernatant (*W*_ds_) were calculated. WAI is the ratio of the weight of swollen starch granules after centrifugation (g) to their dry mass (g):

(2) $$\begin{array}{c} \text{WAI}=\frac{W_{p}}{W_{\text{dp}}}\;\left(\text{g}/\text{g}\right). \end{array}$$

WSI is the ratio of dry mass of solubles in supernatant to the dry mass of whole starch sample (*W*_*s*_):

(3) $$\begin{array}{c} \text{WSI}=\frac{W_{\text{ds}}}{W_{s}}\times 100\;\left(\text{g}/\text{g}\right). \end{array}$$

Similarly, as executed for calculating WA, LSS powder was mixed with three additives and the procedure was continued as it was described for pure starch suspensions.

Water solubility index (WSI) was calculated based on the assumption also mentioned in a study by Mandala and Bayas [13] that the total amount of additives remained in the supernatant. WSI was calculated after subtracting dry weight of acacia gum, NaCl, and sucrose from dry mass of soluble substances in supernatant.

### 2.4. Experimental Design and Statistical Analysis

The experiment was designed according to a rotatable central composite surface response model with three variables and three levels. The three independent variables were acacia gum (0.5–1.5%), salt (0.5–1.5%), and sucrose (10–30%). The three levels were coded as −1, 0, and 1, for the statistical analyses (Table 1). The numbers of design points were obtained using the statistical software package, that is, Design Expert 8.0 (Stat-ease, Inc.) based on the number of independent variables. The additives which influence the physical properties of starch were selected for study. WA, WAI, and WSI were taken as responses. The range of additives levels was selected on the basis of formulations normally used in starch-based breakfast cereals and processed foods [17, 21]. As LSS involved three independent variables, the total number of combinations made was twenty in which the number of design points was 14 with six center point replications. The center points for these designs were selected with additives at levels expected to yield satisfactory results. For each response, analysis of variance (ANOVA) was conducted to determine significant differences among various additive combinations. WA and WAI were maximized and WSI was minimized to obtain the most desirable optimized formulation of additives.

## 3. Results

Effect of three additives on LSS was studied and optimized combination was obtained. The experimental design with three independent variables and the respective responses for the LSS are given in Table 2.

Since WA, WAI, and WSI are important properties to assess the behavior of starch in food system containing water as one of the most essential ingredients, these parameters were taken as responses. The regression analysis of the responses was conducted by fitting 2FI model as suitable for the respective response. Analysis of variance was carried out to assess the significance of hypothesis for selected model and responses (Table 3).

2FI model was highly suitable for WA (*P* < 0.0001), WAI (*P* = 0.0001), and WSI (*P* = 0.0003) response in LSS. The *P* values given in the parenthesis for each response are for the model significance. The *P* value indicates the *P* > *F*-value which should be less than 0.05 for model to be significant; otherwise the model cannot be used for further routing or prediction. All the 2FI models were fitted using Design Expert software. Multiple regression equations as obtained for all the three responses of LSS with actual factors are represented as follows.

*Actual Factors:*

(4) WA=+20.75254+38.18289∗Acacia  Gum−0.42500∗Acacia  Gum∗Sucrose−9.50000∗Acacia  Gum∗NaCl

(5) WAI=+10.65903+5.62732∗Acacia  Gum−0.44882∗NaCl−4.18000∗Acacia  Gum∗NaCl

(6) $$\begin{array}{c} \text{WSI}=+0.25906-0.00419*\text{Sucrose}. \end{array}$$

**Figure 1:**
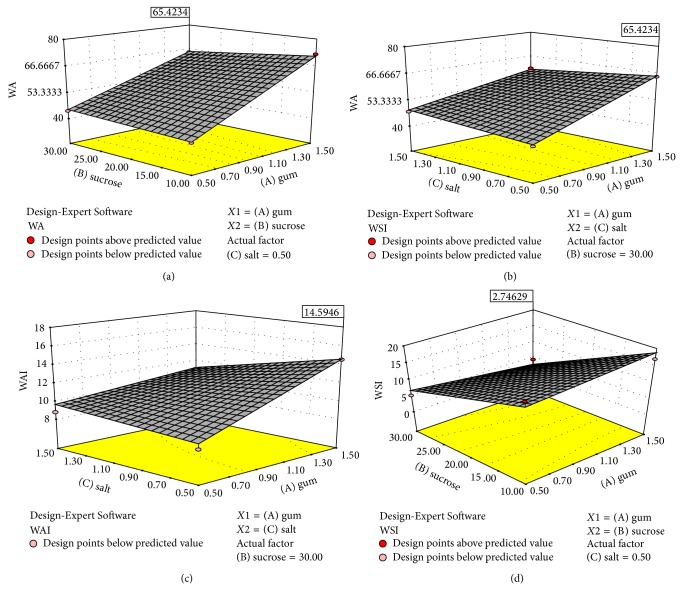
3D plots depicting effect of (a) sucrose (B) and acacia gum (A) on water absorption of lotus stem starch; (b) NaCl (C) and acacia gum (A) on water absorption of lotus stem starch; (c) NaCl (C) and acacia gum (A) on water absorption index; and (d) sucrose (B) and acacia gum (A) on water solubility index of lotus stem starch.

Further, the optimization of variable levels was achieved by desirable maximization or minimization of the necessary response along the fitted 2FI models by numerical optimization procedure of Design Expert software. The effect of change in the levels of selected additives on the response parameters is represented in Figure 1. As can be seen from Table 2, maximum response for WA (72.50%) was obtained with acacia gum—1.50%, sucrose—10%, and NaCl—0.5%. Minimum response for WA (41.00%) was obtained with acacia gum—0.16%, sucrose—20%, and NaCl—1%. Effect of acacia gum on WA of LSS was highly significant (*P* < 0.05) leading to increase in the latter value with increasing content of the former (see (4)). Interaction effect of acacia gum with NaCl and also with sucrose was also found significant (*P* < 0.05) for WA. The coefficient of determination (*R* ^2^) was obtained to be 0.9486 for WA (Table 3). According to Henika [22], coefficients of determination more than 0.75 for constructed models are relatively adequate for prediction. The effects of three independent variables on WA of LSS are depicted in response surface plots (Figures 1(a)-1(b)).

Maximum response for WAI (15.20%) was obtained with acacia gum—1.50%, sucrose—10%, and NaCl—0.50%. Minimum response for WAI (8.30%) was obtained with acacia gum—0.50%, sucrose—30%, and NaCl—0.50%. Acacia gum and NaCl individually and their interaction were found to affect WAI of LSS significantly (*P* < 0.05, see (5)). Gum addition increased WAI, but NaCl demonstrated the reverse role. Interaction of NaCl and acacia gum reduced the WAI value of LSS. A regression equation which had a good fitting capacity for WAI of LSS was constructed with high coefficient of determination (*R* ^2^ = 0.8449). The effects of independent variables on WAI of LSS are depicted in response surface plots (Figure 1(c)).

In case of WSI, maximum response for WSI (19.00%) was obtained with acacia gum—1%, sucrose—3.18%, and NaCl—1%. Minimum response for water solubility index (0.20%) was obtained with acacia gum—1%, sucrose—36.82%, and NaCl—1%. Sucrose was found as significant factor (*P* < 0.05) affecting the value (see (6)). WSI was reduced as the sucrose content increased. High coefficient of determination (*R* ^2^ = 0.8192) for WSI of LSS was obtained.

**Figure 2:**
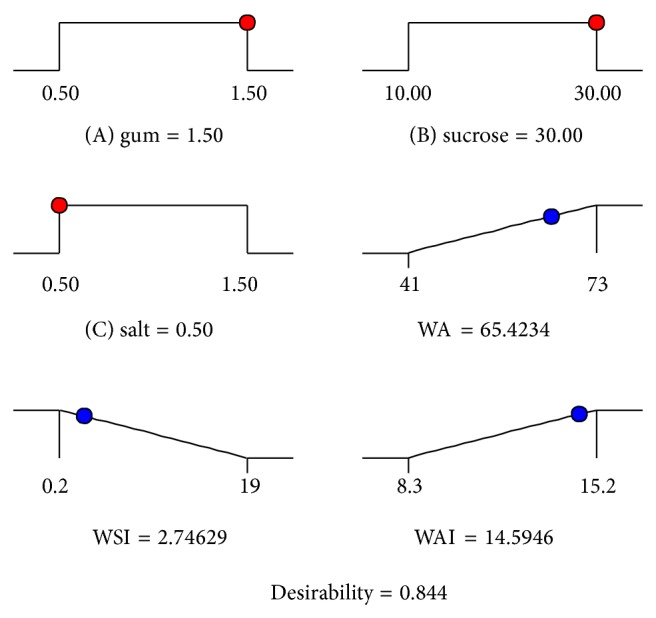
Graphical representation of optimized levels of independent variables of lotus stem starch.

Finally in LSS, the variables were optimized based on the maximization of the WA and WAI and minimization of WSI values. The solutions were required to maximize the desirability function for the given criteria by being at random starting points. The optimized additives levels with suitable desirability are as shown in Figure 2. LSS thus treated with optimized level of additives were verified for the predicted values and the actual values for the responses. Actual and predicted values were compared using Student's *t*-test and the *P* value (<0.05) suggested no significant difference between the two. Hence, the fitted models are best suitable for predicting the responses of the study (Table 4).

## 4. Discussion

For obtaining optimized solution of formulation for the development of appropriate final product, responses were suitably maximized and minimized while performing RSM. WA and WAI of any starch of edible purpose should be high enough for proper gelatinization during heating in the presence of moisture for making it digestible [21]. WSI depicts the ability of starch to leach out amylose from the chains [17]. If this happens then the yield of starch could reduce and also desired property of starch may change [13]. Hence, WSI of starch should be very less. With this view, WA and WAI were kept maximized in RSM and WSI was kept minimized. Among the combinations studied in the present study, the combination which yielded maximum desirability was selected.

### 4.1. Water Absorption (WA)

Water absorption (WA) is the ratio of the wet weight of the sediment starch gel to its dry weight [23]. Water absorption (WA) of starch is an imperative parameter for expressing the interaction of native starch with water in processing of food products to improve yield and consistency and impart desired body and textural characteristics to the food. When unheated native LSS was used to estimate water absorbed by granules, significant increase was observed with increasing acacia gum content, which can be explained by the fact that hydrocolloids promotes swelling. WA of LSS was restricted by the interaction effect of gum with salt and sugars. The reason may be attributed to the limited water availability to starch granule in the presence of sugar and salt [24]. Sucrose addition to starch dispersion significantly decreased water absorption attributed to the fact that sucrose has a higher number of OH groups which makes it more hydrophilic, thus limiting water availability to LSS [16]. Teixeira et al. [16] reported decrease in water uptake (~60%) by cassava starch with sugar (2%) addition. Likewise, Chen et al. [25] also reported significant decrease in swelling of potato starch and flaxseed polysaccharide potato starch (FG-PS) complexes due to the addition of sucrose (2–4%) owing to reduced water activity and formation of more bonding water which was unable to take part in absorption process.

No significant effect of NaCl was observed on WA which can be supported by the study of Zhu et al. [26], which concluded that if no heat is applied, no significant influence of NaCl on the particle size distribution of wheat starch granules was observed. Hence, it can be interpreted that salts cannot be classified as swelling inhibitor or promoter as such, since it is the function of temperature at which the swelling is observed.

### 4.2. Water Absorption Index (WAI) or Swelling Power

Swelling power is simple analytical test to measure the water uptake during the gelatinization of starch. Amylopectin is considered as sole contributor to water absorption and subsequent swelling and pasting of starch granules, whereas amylose tends to retard this phenomenon [27]. On heating at 90°C for 30 min, significant changes in water absorption index (WAI) or swelling power of LSS were administered by addition of acacia gum and NaCl and by interaction thereof. Effect of additives was investigated on gelatinized starch existing in two forms, that is, continuous phase (amylose/amylopectin matrix) and the dispersed phase (starch granules). Heating of starch dispersion causes swelling of granules, which influences the properties of both continuous and dispersed phases [28]. Increase in WAI with acacia gum addition can be supported by the study of Mandala and Bayas [13] which reported that xanthan addition (0.09%) to wheat starch enhanced swelling of starch dispersion (2%) during heating at 90°C for 30 min. The following assumption about the role of gums in starch systems supports the finding of Mandala and Bayas [13]. According to Abdulmola et al. [29], starch molecules can interact and a network can be created at concentrations well below “close-packing.” Xanthan promotes adhesive interactions and entraps the gelatinized granules by keeping them close. This leads to force enhancement applied to them, facilitating swelling and amylose solubilisation and its exudation. Further increase in temperature causes leaked amylose and the xanthan in the continuous phase to create a film around the granules which further inhibit swelling. Effect of hydrocolloids addition on swelling of starch granules during heating was also reported by Chaisawang and Suphantharika [21] suggesting that addition of guar or xanthan gum (0.073%) slightly enhanced swelling of native tapioca starch (1.25%) granules at temperatures that ranged from 60 to 90°C.

Decrease in WAI of LSS with increasing NaCl content occurred due to competition between salts and starch for available water molecules at high temperatures [14]. Also, electrostatic interaction between starch and ions from NaCl has ability to limit the swelling of starch granules [30]. Similarly, Zhu et al. [26] reported limited promoting effect of NaCl on wheat starch at higher temperatures concluding that swelling inhibiting effect of NaCl is largely temperature dependent. Significant decrease in swelling power of rice starch subjected to heating at 75°C on addition of 0.1 M NaCl solution further sustains the results [14].

WAI of LSS decreased due to interaction between acacia gum and NaCl. Similar influence of hydrocolloid and salt on swelling power of rice starch has been reported by Samutsri and Suphantharika [14]. Addition of salts (0.1 M) significantly decreased water uptake of rice starch complexed with xanthan and guar gum in 19 : 1 (w/w) ratio of starch and gum. Hence, conclusion can be drawn that effect of NaCl on reduction of swelling power of rice starch follows the order of the Hofmeister series which is a classification of ions in order of their ability to salt-out or salt-in proteins [14].

### 4.3. Water Solubility Index (WSI)

WSI of LSS declined with increasing sucrose content. Presence of sucrose inhibits the degradation of the starch molecules leading to lower WSI [31]. Spies and Hoseney [32] proposed that stabilization of amorphous region of the starch granules occurs when sugar molecules possibly restrict the mobility and flexibility of starch chains by forming bridges with more starch chains. This kind of sugar-starch chain interaction could be the reason for observed reduced amylose leaching. Richardson et al. [33] conducted a study to investigate the effect of sucrose (12 or 24%, w/w) on solubility index of wheat starch dispersion (8%) which revealed delayed amylose leakage and granule fragmentation above 50°C.

## 5. Conclusion

In the presence of acacia gum, NaCl, and sucrose, the physical properties of lotus stem starch might be governed by hydrocolloid-salt, hydrocolloid-sugar interaction and individual effect of these additives. Desired or optimum level of these additives was obtained by using RSM, which returned 1.5% of acacia gum, 0.5% of NaCl, and 30% of sucrose with a desirability of 0.844. WA, WAI, and WSI are 65.42%, 14.95, and 2.74%, respectively. In presence of acacia gum, swelling power of starch increased. NaCl cannot be classified as swelling inhibitor or promoter as such, since it is the function of temperature at which the swelling is observed. Sucrose was found to inhibit water absorption by limiting the water availability to starch in the presence of acacia gum, thus reducing the swelling promoting effect of the latter. Sucrose also retarded amylose leaching (WSI) by mechanism of building bridges with starch chains and reducing their flexibility. Hence, results of present investigation suggest important practical inferences in applications of assayed additives in starch-based food products containing lotus stem starch as one of the major ingredients which is highly popular as functional food in east Asian part of the world.

## Figures and Tables

**Table 1:** Experimental ranges and levels of independent variables used in RSM in terms of actual and coded factors.

	Variables	−1	0	1
Acacia gum (%, w/w)	A	0.5	1.0	1.5
Sucrose (%, w/w)	B	10	20	30
Salt (%, w/w)	C	0.5	1.0	1.5

**Table 2:** Experimental design depicting levels of additives and their responses in lotus stem starch.

Run	Factor 1 *A*: Gum (%)	Factor 2 *B*: Sucrose (%)	Factor 3 *C*: Salt (%)	Responses		
				WA	WAI	WSI
1	1.00	20.00	1.00	62.00	10.10	15.00
2	1.00	20.00	1.00	57.50	11.05	12.00
3	1.84	20.00	1.00	73.00	13.35	13.00
4	1.50	10.00	0.50	72.50	15.20	17.00
5	1.50	30.00	0.50	65.00	14.52	04.30
6	1.50	10.00	1.50	66.00	09.30	16.00
7	0.50	10.00	0.50	42.00	10.30	18.00
8	0.50	30.00	0.50	44.00	08.30	05.20
9	0.50	10.00	1.50	46.00	08.70	03.00
10	1.00	36.82	1.00	54.00	11.78	00.20
11	1.00	03.18	1.00	55.00	12.76	19.00
12	1.00	20.00	1.00	53.00	12.95	11.00
13	1.00	20.00	1.84	53.00	09.92	11.00
14	1.00	20.00	1.00	53.00	11.25	09.00
15	1.50	30.00	1.50	60.00	10.95	00.60
16	1.00	20.00	1.00	53.50	11.20	09.00
17	1.00	20.00	0.16	56.00	13.45	10.00
18	0.16	20.00	1.00	41.00	09.88	08.00
19	1.00	20.00	1.00	54.00	11.82	09.50
20	0.50	30.00	1.50	47.50	08.79	00.50

**Table 3:** ANOVA and model statistics of lotus stem starch.

S. number	Term	Response		
		Water absorption (WA)	Water absorption index (WAI)	Water solubility index (WSI)
1	Model	2FI	2FI	2FI
2	*F* value	39.97	11.80	9.82
3	*P* > *F*	<0.0001	0.0001	0.0003
4	Mean	55.40	11.28	9.57
5	Standard deviation (SD)	2.49	0.93	3.01
6	CV%	4.50	8.24	31.47
7	*R* squared	0.9486	0.8449	0.8192
8	Adjusted *R* squared	0.9232	0.7733	0.7358
9	Predicted *R* squared	0.9232	0.5097	0.2046
10	Adequate precision	23.033	11.839	10.391

**Table 4:** Predicted response versus actual response.

Values	Response		
	WA	WAI	WSI
Predicted	65.42	14.59	2.75
Actual	64.66	14.46	2.68

## References

[1] Wang Q. C., Zhang X. Y. (2004). *Lotus Flower Cultivars in China*.

[2] Ono Y., Hattori E., Fukaya Y., Imai S., Ohizumi Y. (2006). Anti-obesity effect of *Nelumbo nucifera* leaves extract in mice and rats. *Journal of Ethnopharmacology*.

[3] Ling Z.-Q., Xie B.-J., Yang E.-L. (2005). Isolation, characterization, and determination of antioxidative activity of oligomeric procyanidins from the seedpod of Nelumbo nucifera Gaertn. *Journal of Agricultural and Food Chemistry*.

[4] Lee H. K., Choi Y. M., Noh D. O., Suh H. J. (2005). Antioxidant effect of Korean traditional lotus liquor (Yunyupju). *International Journal of Food Science and Technology*.

[5] Mukherjee P. K., Saha K., Pal M., Saha B. P. (1997). Effect of Nelumbo nucifera rhizome extract on blood sugar level in rats. *Journal of Ethnopharmacology*.

[6] Liu C.-P., Tsai W.-J., Lin Y.-L., Liao J.-F., Chen C.-F., Kuo Y.-C. (2004). The extracts from Nelumbo nucifera suppress cell cycle progression, cytokine genes expression, and cell proliferation in human peripheral blood mononuclear cells. *Life Sciences*.

[7] Sridhar K. R., Bhat R. (2007). Lotus—a potential nutraceutical source. *Journal of Agricultural Technology*.

[8] Singh N., Singh J., Kaur L., Sodhi N. S., Gill B. S. (2003). Morphological, thermal and rheological properties of starches from different botanical sources. *Food Chemistry*.

[9] Xu S.-Y., Shoemaker C. F. (1986). Gelatinization properties of Chinese water chestnut starch and lotus root starch. *Journal of Food Science*.

[10] Man J., Cai J., Cai C., Xu B., Huai H., Wei C. (2012). Comparison of physicochemical properties of starches from seed and rhizome of lotus. *Carbohydrate Polymers*.

[11] Gani A., Masoodi F. A., Wani S. M. (2013). Characterization of lotus stem (*Nelumbo nucifera*) starches purified from three lakes of India. *Journal of Aquatic Food Product Technology*.

[12] Davidou S., Le Meste M., Debever E., Bekaert D. (1996). A contribution to the study of staling of white bread: effect of water and hydrocolloid. *Food Hydrocolloids*.

[13] Mandala I. G., Bayas E. (2004). Xanthan effect on swelling, solubility and viscosity of wheat starch dispersions. *Food Hydrocolloids*.

[14] Samutsri W., Suphantharika M. (2012). Effect of salts on pasting, thermal, and rheological properties of rice starch in the presence of non-ionic and ionic hydrocolloids. *Carbohydrate Polymers*.

[15] Maache-Rezzoug Z., Bouvier J.-M., Allaf K., Patras C. (1998). Effect of principal ingredients on rheological behaviour of biscuit dough and on quality of biscuits. *Journal of Food Engineering*.

[16] Teixeira E. M., da Róz A. L., Carvalho A. J. F., Curvelo A. A. S. (2007). The effect of glycerol/sugar/water and sugar/water mixtures on the plasticization of thermoplastic cassava starch. *Carbohydrate Polymers*.

[17] Jin Z., Hsieh F., Huff H. E. (1995). Effects of soy fiber, salt, sugar and screw speed on physical properties and microstructure of corn meal extrudate. *Journal of Cereal Science*.

[18] Wei C., Qin F., Zhu L. (2010). Microstructure and ultrastructure of high-amylose rice resistant starch granules modified by antisense RNA inhibition of starch branching enzyme. *Journal of Agricultural and Food Chemistry*.

[19] Beuchat L. R. (1977). Functional and electrophoretic characteristics of succinylated peanut flour protein. *Journal of Agricultural and Food Chemistry*.

[20] Leach H. W., McCowen L. D., Schoch T. J. (1959). Structure of the starch granule. I. Swelling and solubility patterns of various starches. *Cereal Chemistry*.

[21] Chaisawang M., Suphantharika M. (2006). Pasting and rheological properties of native and anionic tapioca starches as modified by guar gum and xanthan gum. *Food Hydrocolloids*.

[22] Henika R. G. (1982). Use of response-surface methodology in sensory evaluation. *Food Technology*.

[23] Crosbie G. B. (1991). The relationship between starch swelling properties, paste viscosity and boiled noodle quality in wheat flours. *Journal of Cereal Science*.

[24] Ahmad F. B., Williams P. A. (1999). Effect of salts on the gelatinization and rheological properties of sago starch. *Journal of Agricultural and Food Chemistry*.

[25] Chen H.-H., Wang Y.-S., Leng Y., Zhao Y., Zhao X. (2014). Effect of NaCl and sugar on physicochemical properties of flaxseed polysaccharide-potato starch complexes. *ScienceAsia*.

[26] Zhu W. X., Gayin J., Chatel F., Dewettinck K., van der Meeren P. (2009). Influence of electrolytes on the heat-induced swelling of aqueous dispersions of native wheat starch granules. *Food Hydrocolloids*.

[27] Tester R. F., Morrison W. R. (1990). Swelling and gelatinization of cereal starches. I. Effects of amylopectin, amylose, and lipids. *Cereal Chemistry*.

[28] Li J.-Y., Yeh A.-I. (2001). Relationships between thermal, rheological characteristics and swelling power for various starches. *Journal of Food Engineering*.

[29] Abdulmola N. A., Member M. W. N., Richardson R. K., Morris E. R. (1996). Effect of xanthan on the small-deformation rheology of crosslinked and uncrosslinked waxy maize starch. *Carbohydrate Polymers*.

[30] Oosten B. J. (1990). Interactions between starch and electrolytes. *Starch/Stärke*.

[31] Sopade P. A., Le Grys G. A. (1991). Effect of added sucrose on extrusion cooking of maize starch. *Food Control*.

[32] Spies R. D., Hoseney R. C. (1982). Effect of sugars on starch gelatinization. *Cereal Chemistry*.

[33] Richardson G., Langton M., Bark A., Hermansson A.-M. (2003). Wheat starch gelatinization—the effects of sucrose, emulsifier and the physical state of the emulsifier. *Starch*.

